# The Earthy Phosphates

**Published:** 1885-09

**Authors:** W. C. Barrett


### Independent Practitioner.

Vol. VI. September, 1885. No. 9.

### Original Communications.

### THE EARTHY PHOSPHATES.

Abstract of a Paper Read Before the American Dental Association at Minneapolis, August, 1885.

BY DR. W. C. BARRETT.

The administration of the earthy phosphates to pregnant women and to young children has been a favorite prophylactic method of treatment with many intelligent dentists. The theory upon which this system was founded is, that decayed or defective teeth owe their condition to trophic disturbances, and that it is but necessary to supply the elements missing to produce perfect dentures. This theory is a very plausible one, and it gives loquacious practitioners excellent opportunity to enlarge upon the wonderful process of gestation, and the marvelously interesting double function of the expectant mother, whose digestive apparatus must furnish, not only pabulum to sustain her own physical being during a trying period, but sustenance to the fœtal man or woman which she carried beneath her heart. If her own teeth decayed during this period, because of the neglect which at such times is common, she was perhaps treated to long dissertations upon the imperative demands of the growing contents of her uterus, which, not finding the earthy elements needed in the blood with which she supplied it, was robbing her own osseous system to supply its wants. If her teeth were found to be soft, it was because their character had changed, and the lime salts of their crystalline structure had been taken out to build up the young child. Corroborative evidence, when looked for with biased judgment, was not lacking, and dentists had many tales to relate of the most stupendous changes effected in the dental development of children through a judicious administration of the lacto-phosphate of lime to the mother during pregnancy. The instances in which such results seemed apparent were cited, while those in which no effect resulted were not included in the category, or were attributed to a want of faithfulness in taking the prescription.

We believe that which we desire to believe, and there is no difficulty in finding apparent confirmatory evidence to sustain the most absurd of postulates, when one sets out with a determination to do so.

There is probably no one who has followed the prescribing of the earthy phosphates for a supposed dystrophic condition, who will not, if his memory be sufficient and his data perfect, call to recollection very many complete failures. If he shall have traced the after-history of children born of mothers who, during pregnancy, were subjected to the phosphatic treatment, he will probably find quite as many with defective teeth as in those who were born under other conditions.

Earlier in my own professional history, I experimented with the different preparations in numerous cases. One of the first of these was that of a lady pregnant with her second child, the first being in a deplorable dental state. The seeming results were surprising. Not only was her own gestation much more pleasant and easy, but the dentition of her infant was almost entirely without the usual febrile disturbances, and the child's teeth, up to the time when I lost sight of him through the removal of the parents, presented a remarkable contrast to those of his elder brother. This case would have possibly confirmed me in the use of the phosphates, were it not that about the same time I had contrary experiences.

In one notable case, that of a lady pregnant with her fifth child, I persuaded her to a thorough course of my then favorite remedy. All the other children had excellent teeth, much above the average. There was no special reason why she should be subjected to prophylactic treatment, except that I thought I saw disastrous effects from her condition upon her own teeth, and because I was pushing the investigation with strong hopes that I had hit upon means by which a perfect dentition might be assured to every child. As time rolled on I beheld the direct antithesis of the first cited case. The child had all manner of difficulty in getting its teeth, and when they were in place I saw, to my confusion, the only really bad dentition in the family. Of all the women whom I subjected to this treatment, there was not one in which the experience of the lady of the first instance was repeated. Experience alone led me to entirely abandon the practice, and when, subsequently, I made a more thorough study of the physiology of nutrition, I became confirmed in my skepticism concerning the utility of the feeding of phosphates to pregnant women, or even the indiscriminate recommending of such foods as are particularly rich in earthy materials. The facts are against it.

A few years since it was common to hear denunciations of the use of fine flour, from which it was declared that the miller had eliminated all that was of use in the building up of the bony system. Elaborate papers have been read before this Association, in which it was demonstrated to the satisfaction of the really thoughtful and honest essayist, that the decay of teeth, which was supposed to be a modern disease, was due to the lack of phosphates in the fine flour that formed the chief article of subsistence. Since then, computations have been made of the amount of bone-making material that is found in the finest of wheat flour, and of the amount that is needed by both mother and fœtus during the period of gestation, and it has been demonstrated that should she subsist entirely upon this article of diet, there would still be an excess of the lime salts. It is a fact that during pregnancy there is almost universally a continual elimination of these principles, which are easily traced in the excretions. Any man, or pregnant woman, who lives upon almost any diet that is sufficient to sustain life, will, if the nutritive organs be in proper condition, find more than is necessary of these elements to keep the osseous system in good condition. It must be remembered that the nutritive changes in the bones and teeth are less than in the other tissues of the body, because they are more permanent in character and structure. Especially is this the case with the teeth, in which the trophic changes are very limited indeed. That such a dystrophy may exist as shall materially affect these organs, no one will probably deny; but the process will of necessity be but a slow one, and the changes will not soon be manifest.

And now let me detail some of the physiological reasons why the giving of the earthy phosphates for nutritive purposes must be a mistaken treatment, and why, to my conception, it is founded upon erroneous assimilative views.

All pabulum must originally be derived from the earth. That is the primal source of all nutritive material. But there is no order or class in the animal kingdom that can elaborate it. There is no animal organism that can derive nourishment directly from earthy matter. That function rests solely in the vegetable kingdom. Animals are not primal organizers. They cannot digest the inorganic. They require organic structures for their food. But the study of vegetable physiology shows that they have the ability to assimilate inorganic matter, and out of earthy matter to organize tissue that may serve for the food of the higher orders. When matter has once been organized into vegetable products, it may serve for the sustenance of animals. Some of the animal kingdom subsist solely upon matter but once removed from the inorganic. To this class belong the graminivora. Others of the animal kingdom require that their food shall have been twice organized; first from the earth into a vegetable form, and again by an animal into a higher form. To this class belong the carnivora, who cannot digest or assimilate vegetable organisms until they shall have been reorganized into an animal.* Others are omnivorous, and their digestive apparatus will prepare for nutrition matter that has been but once organized into vegetable life, or that has been again organized into animal existence. To this class belongs man. But neither he nor the graminivora can make nutritive use of inorganic matter, any more than can the carnivora. It follows, then, that if inorganic matter be introduced into an animal organism, it is entirely foreign, and must be eliminated in an unchanged condition. If it remain within the system, it is essentially and must remain a foreign substance, an irritant that, if not promptly ejected, will create internal disturbances of a more or less serious nature. All inorganic matter, then, is foreign to the animal system, and so far as nutrition goes it is not only entirely useless, but absolutely mischievous.

Some of the proximate principles of animal bodies are made up mainly of inorganic material, but they never exist as simple substances, unless we may call the iron of the blood a principle. That

* It is a rather singular fact that most of the animals that require their food twice organized are unfit for food themselves. Their flesh is rank, unpalatable and innutritious. There are exceptions among fishes and birds, but of the mammalia, the flesh-eaters are themselves uneatable.

exists only in a kind of solution, held there by the other constituents, and it is not assimilated directly. The calcium of the bones and teeth exists in combination with other substances, and it is never assimilated directly, but it is elaborated, and the combination formed within the system. Carbonate of lime and the phosphate of magnesia are not taken up as such, but the carbon, the phosphorus, the calcium, and the oxygen are elaborated within the organism, and their chemical union is there completed when they are built into the tissues. The building of this animal house of ours cannot be brought about by feeding bricks and mortar. The raw materials must be furnished in other compounds, which it is the province of the nutritive apparatus to disorganize, to separate into their constituent elements, and to recombine into tissues. Every particle of tissue principle must be elaborated within the body, and built up, not from compounds, but from simple elements. If carbonate of lime be needed for the teeth, it is of no use to feed oyster-shells. The system will not take carbonate of lime, but it will elaborate the materal from the calcium and carbon and oxygen, which it derives from food, and it will take its carbonate of lime in no other way. It is the same with the phosphates, and hence the inutility of giving any preparation of that material, which will not serve as pabulum. So complete and perfect is this nutritive process in the healthy organism, and so universally and admirably are the elements provided in all organic matter, that a perfect digestion will find in any material that is fit for food, sufficient of the different ingredients to elaborate into pabulum for all the tissues.

Were not this the case, it would be impossible for the different races to exist under all the varying conditions in which they must live. The dweller in the hyperborean regions, where vegetable life scarcely exists, must make a subsistence almost exclusively out of an animal diet. But his whole system is as well nourished as is the omnivorous dweller in the temperate regions. There are those who live upon an exclusively vegetable diet, and none of the tissues are starved. Existence has been prolonged exclusively upon fruits, yet every organ was perfect, because of this universal diffusion of the essential elements of nutrition. The simple substances from which the compound tissues of the body are composed are comparatively few, and are found everywhere. Were it otherwise; were it the case that the organism was unable to elaborate its compounds from the elementary substances; were it the fact that carbonate of lime, and fluoride of calcium, and all the other compounds must be supplied as they exist, it can readily be seen that animals could not subsist upon a simple diet. It would be necessary to supply such foods as contain the exact compounds needed, and this, except under the most favorable conditions, would be impossible. Hence, but a very small proportion of the earth's surface would be habitable, and most orders of animals would soon become extinct through inability to obtain the exact compounds needed for so complex a nutrition. The laws governing our existence are simple, if we would but consider them intelligently. Animal life can subsist upon almost any kind of organic matter, not absolutely poisonous, and yet be well nourished.

Inorganic matter does, however, play an important part in the human economy, but man is the only animal that makes any extended use of it. Many inorganic materials act as special irritants or excitants to definite organs. Their very presence in the system may induce certain structural or functional changes, and thus in abnormal conditions they may play a useful part. When they are administered for any such purpose we call them remedies, but man is the only animal that makes such use of the inorganic world. Our medical pharmacopœia is largely made up of inorganic matters, and they are given to induce certain changes corrective of others, brought about by dystrophic conditions. If intestinal function ceases through the presence of innutritive or indigestible matter, an inorganic remedy may, by its presence, induce such violent peristaltic action as to eliminate the obstructive matter. Alterative effects follow the ingestion of some inorganic compounds, but it should always be remembered that such inorganic substances are entirely foreign to the organism, and are always extruded at the earliest opportunity. They form no part of the nutrition, and are never built up into the tissues.

It must follow, then, that the giving of the inorganic earthy phosphates for nutritive purposes is always a mistake. If they act at all, it must be remedially, and if they are used they should be intelligently prescribed, like any other agent, and only for their medicinal properties. I have no knowledge that they have any very decided medicinal virtues, and therefore I can see no excuse for dispensing such inert compounds.

